# A Novel Percutaneous Technique for Aorto-Iliac Thrombectomy without the Risk of Embolization

**DOI:** 10.3390/bioengineering10070778

**Published:** 2023-06-29

**Authors:** Rosalinda D’Amico, Thomas Wolff, Sabine Richarz, Lorenz Gurke, Andrej Isaak, Edin Mujagic

**Affiliations:** 1 University Hospital of Basel, Spitalstrasse 21, 4031 Basel, Switzerland; rosalinda.damico@ksa.ch (R.D.); thomas.wolff@usb.ch (T.W.); sabinejohanna.richarz@usb.ch (S.R.); lorenz.guerke@usb.ch (L.G.); 2 Kantonsspital Aarau, Tellstrasse 25, 5001 Aarau, Switzerland; andrej.isaak@ksa.ch; 3 Faculty of Medicine, University of Basel, Klingelbergstrasse 61, 4056 Basel, Switzerland

**Keywords:** endovascular, percutaneous thrombectomy, protection from embolization

## Abstract

Classic surgical thrombectomy of the aorta and iliac arteries through an incision in the groin vessels harbors the risk of embolization to the viscero-renal as well as hypogastric arteries, while percutaneous endovascular thrombectomy techniques can lead to peripheral embolization to the lower limbs. Therefore, we describe a novel, percutaneous technique that tackles the above issues. Furthermore, we also present our initial experience using the technique. The principle of the technique is to percutaneously place large-bore sheaths in the iliac arteries that deliberately occlude the latter to protect the lower limbs from embolization. Through one of these sheaths, over wire Fogarty^®^ catheters can be placed and inflated in the ostia of the coeliac trunk, superior mesenteric artery, renal arteries, and hypogastric arteries as needed. A large thrombectomy balloon catheter is then used to bring any aorto-iliac thrombus into the sheaths, whereafter the thrombus is removed from the sheaths by simply deflating their valves. Additional endovascular procedures of the aorto-iliac branches can be performed as needed. We report nine procedures in 8 patients (4 males and 4 females) with a median age of 63 (53–68.5). Additional endovascular procedures were performed in 6 (66.7%) procedures. All but one procedure were technically successful, and all patients had palpable foot pulses on completion of the procedures, while no patient had clinical signs of peripheral embolization. This technique is a very valid addition to the vascular surgeon’s armamentarium when treating aorto-iliac thrombotic events because it is minimally invasive while still protecting against embolization and offering the flexibility to perform a wide range of additional endovascular procedures where needed.

## 1. Introduction

Since the start of the COVID-19 pandemic, COVID-19 infections have repeatedly been reported to be associated with thrombotic events [1]. These events include appositional thrombi localized in the thoraco-abdominal aorta and iliac arteries, at times even leading to complete occlusion of these vessels [2,3,4,5,6,7,8,9,10,11,12,13,14,15,16,17,18].

Local open thrombectomy through direct access to the thoraco-abdominal aorta has been described as a treatment option [19], but it is highly invasive and not ideal or even unfeasible in potentially very sick patients with COVID-19 pneumonia and respiratory impairment.

Since its introduction in 1962 by Fogarty [20], surgical thrombectomy using inflatable balloon catheters has been the standard approach to treating acute or chronic thrombotic vascular occlusions. These procedures are classically performed through a cutdown and arteriotomy of the access vessels, typically in the groin. However, this open approach through an arteriotomy distant to the site of thrombus harbors the risk of embolization to the aorto-iliac branches between the thrombus and the groins, i.e., the coeliac trunk, superior and inferior mesenteric, renal, and hypogastric arteries, during the thrombectomy maneuvers. To overcome this problem in cases of thrombus localized in the descending thoracic aorta, it has been proposed to simply cover it with a thoracic stentgraft rather than removing it [19]. However, with an extensive thrombotic burden, passing the thrombus with guide wires and the introduction system could again lead to embolization before the stentgraft can be deployed. Furthermore, for thrombus localized in the visceral segment of the aorta, this technique is not possible because the viscerorenal branches would be covered. For these reasons, the only solution in such cases would be to protect the individual branches with balloon catheters before performing the actual thrombectomy. Inserting several wires and catheters that are needed to do so through an arteriotomy in the groin is not possible because it would lead to continuous bleeding through the arteriotomy, and the operator’s hands would not be properly protected from the X-rays needed to guide the procedure because they would constantly be used in the groin to control bleeding.

Minimally invasive percutaneous thrombectomy techniques using modern endovascular devices such as the AngioJet^®^ [21] or Indigo device [22] have been described but have so far not become standard practice. This is likely because such techniques are associated with the potential for peripheral embolization of fragmented particles of thrombus in the lower limbs.

To overcome the above-mentioned problems of both classical balloon thrombectomy and modern endovascular procedures, we developed a novel, minimally invasive technique that tackles the above issues when performing complex aorto-iliac thrombectomy maneuvers by combining old and new techniques. Furthermore, our technique allows for additional endovascular procedures of the viscero-renal aortic branches and hypogastric arteries. We also present a series of the first patients treated using this novel technique.

## 2. Materials and Methods

### 2.1. Patients

All patients treated using the novel technique were routinely entered prospectively into a database. Ethics committee approval has been obtained from the Ethikkommission Nordwest- und Zentralschweiz under the identifier 2021-02139.

### 2.2. Procedural Technique

For this novel technique, unilateral or bilateral approaches are possible as needed. The common femoral arteries are punctured retrogradely under ultrasound guidance. Two Perclose Proglide™ sutures (Abbott, Abbott Park, IL, USA) per access vessel are applied. In situations where femoro-popliteo-cruro-pedal thrombectomy is needed in addition, the femoral bifurcations are exposed by an open standard groin approach, the vessels are punctured directly, and the puncture sites are closed by standard sutures at the end of the procedure. An 8F sheath is placed. A stiff wire is advanced up to the aorto-iliac axes. 8F sheaths are then removed and large bore sheaths (Gore^®^ Dryseal, W. L. Gore & Associates, Inc., Flagstaff, AZ, USA) are inserted. When the thrombus to be removed is localized in the common iliac arteries, care is taken not to dislodge it while inserting the sheath. In practice, the tip of the dilator is never advanced across the thrombus. The size of the sheaths is chosen based on the diameter of the external iliac arteries. In practice, we deliberately choose the outer diameter of the sheath to be slightly bigger than the inner diameter of the external iliac arteries, so the sheaths occlude the vessels and therefore prevent thrombus from embolizing distally during thrombectomy. Guide wires are inserted in the hypogastric arteries, and over-the-wire (OTW) Fogarty^®^ catheters (Edwards Lifesciences, Irvine, CA, USA) are advanced and inflated in the respective ostia to protect them from emboli. The size of the OTW Fogarty^®^ catheters (3F–5.5F) is chosen based on the size of the vessels to be protected. The size of the guide wires is chosen based on the size of the OTW Fogarty^®^ catheters (0.018″ for 3F and 4F catheters; 0.035″ for 5.5F catheters). In patients whose common iliac arteries and external iliac arteries are similar in diameter and not calcified, sheaths are advanced into the common iliac arteries, so balloon protection of the hypogastric arteries is not necessary. For thrombus localized above or in the viscero-renal segment, 0.018″ wires are inserted in each aortic branch, and 3F over-the-wire (OTW) Fogarty^®^ catheters (Edwards Lifesciences, Irvine, CA, USA) are advanced and inflated in all respective ostia. Due to the large diameters of the sheaths, several OTW Fogarty^®^ catheters can easily be inserted through one sheath.

Once the sheaths and all necessary OTW Fogarty^®^ catheters are in place, thrombectomy maneuvers can be performed as needed, either using standard Fogarty^®^ catheters or OTW Fogarty^®^ catheters. Furthermore, when any aortic branches are already occluded by thrombus, rather than protecting those branches using OTW Fogarty^®^ catheters they can be thrombectomized using these same catheters. The thrombus is then removed from the DrySeal sheaths by simply deflating their valves and flushing the thrombus out. After removal of the sheaths, puncture sites are closed using Perclose Proglide™ sutures.

### 2.3. Endpoints

This is a feasibility study. Endpoints are therefore limited to technical and clinical success. Technical success is defined as complete thrombectomy without the need for an unplanned conversion to open surgery. Clinical success is defined as the resolution of either the limb threatening ischemia or the claudication that required the procedure.

### 2.4. Statistical Analysis

Due to the small number of cases, statistical analysis is purely descriptive. For continuous variables, median values, interquartile ranges (IQR), and ranges are given, while percentages are given for categorical variables.

## 3. Results

A total of nine procedures were performed on eight patients. Patient and procedural characteristics are summarized in Table 1.

### 3.1. Patient 1

**Figure 1:**
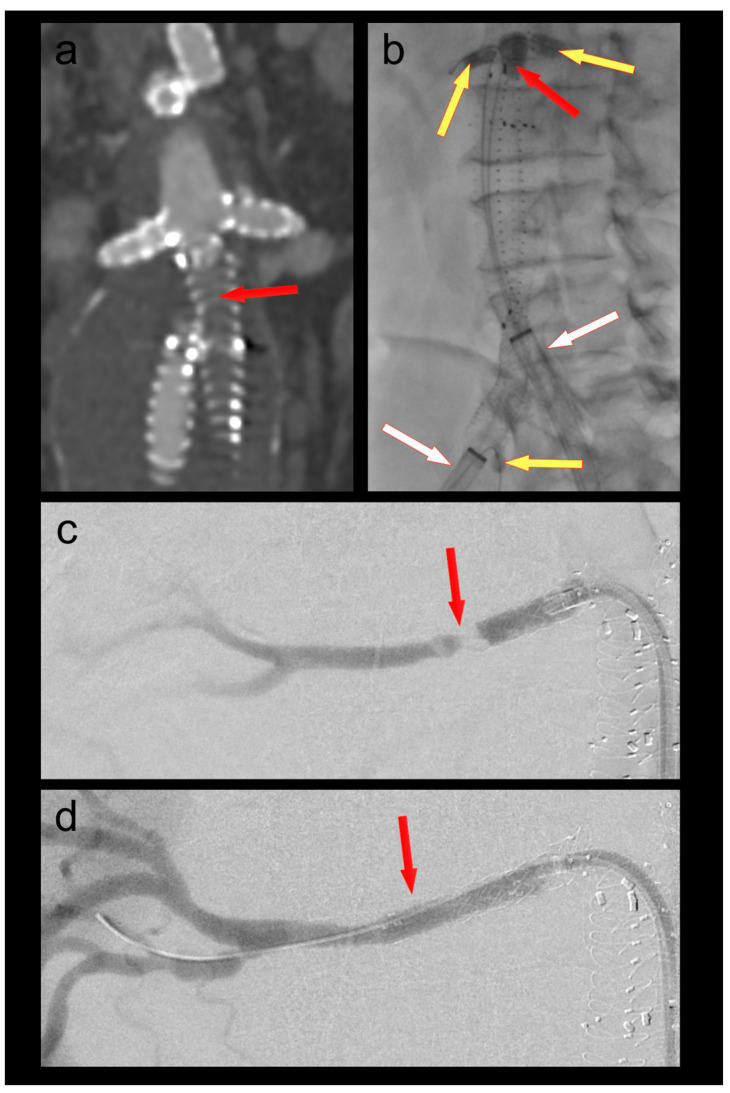
(**a**) Thrombotic occlusion of the right EVAR limb (ballerina situation) (red arrow); (**b**) thrombectomy of the graft limb (red arrow) under balloon protection of both renal bridging stentgrafts and the right hypogastric artery (yellow arrows). The large-bore sheaths are occlusive in the left graft limb and in the right external iliac artery (white arrows); (**c**) Iatrogenic dissection of the right renal artery (red arrow); (**d**) Right renal artery after implantation of a self-expanding stent graft (red arrow).

A 70-year-old male patient who had undergone a 4-fenestration endovascular aneurysm repair (EVAR) 6 months prior was admitted with new-onset short-distance claudication of his right leg. CT angiography revealed a complete occlusion of the right EVAR limb and a non-occlusive thrombus in the left limb. The renal fenestrations/origins of the renal bridging stentgrafts were only a few millimeters above the graft bifurcation (Figure 1). Both renal bridging stentgrafts and the right hypogastric artery were protected using 3F OTW Fogarty^®^ catheters over 0.018″ guide wires. The left hypogastric artery was protected by advancing the sheath into the distal EVAR limb. A thrombectomy of both limbs was performed using a 6F Fogarty^®^ catheter (Figure 1). An iatrogenic dissection of the right renal artery at the distal end of the bridging stent graft was treated by implantation of a 5 × 25 mm Gore^®^ Viabahn self-expandable stent graft. Postoperative CT-scan showed complete thrombectomy of both limbs and ruled out any embolization of the renal and hypogastric arteries (Figure 1).

The same patient was treated a second time one year later using the same technique for an occlusion of the contralateral EVAR limb.

Foot pulses were palpable bilaterally after both procedures.

### 3.2. Patient 2

**Figure 2:**
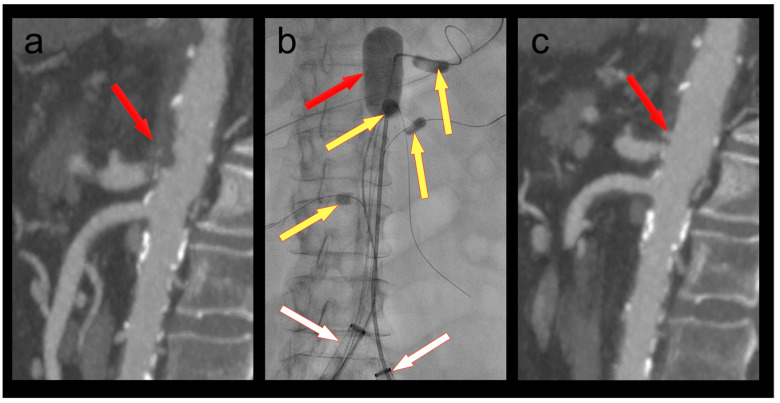
(**a**) Thrombus in the aorta and in the origin of the coeliac trunk (red arrow); (**b**) thrombectomy (red arrow) of the thoracoabdominal aorta under balloon protection of the coeliac trunk, superior mesenteric artery, and both renal arteries (yellow arrows). The large-bore sheaths are occlusive in both common iliac arteries (white arrows); (**c**) Postoperative CT scan with no residual thrombus in the aorta or the coeliac trunk (red arrow).

A 61-year-old female patient was admitted because of respiratory failure due to bilateral COVID-19 pneumonia. A CT angiography detected several appositional thrombi throughout the thoracoabdominal aorta, the biggest of which was located at the origin of the coeliac trunk (Figure 2). Another scan two days later showed progression of the thrombi in spite of therapeutic heparin. The coeliac trunk was nearly occluded, and the right hepatic artery was completely occluded. Furthermore, the patient had acute cholecystitis. To avoid further embolization, we decided to thrombectomize the aorta and the origin of the coeliac trunk. The coeliac trunk, superior mesenteric artery, and both renal arteries were protected by OTW Fogarty^®^ catheters, while the hypogastric arteries were protected by advancing the large-bore sheaths into the common iliac arteries. Thrombectomy of the entire aorta into the large sheaths was performed using an 8–22F Fogarty^®^ occlusion catheter (Figure 2). The 5.5F Fogarty catheter that blocked the coeliac trunk was then used to thrombectomize its origin. There was no relevant residual thrombus in the aorta or the coeliac trunk on completion angiography (Figure 2). No further thrombotic complications were seen under therapeutic anticoagulation, and the patient was discharged home after several months in the hospital.

### 3.3. Patient 3

A 46-year-old male patient with COVID-19 pneumonia was transferred from a peripheral hospital with acute-onset bilateral rest pain and paresthesia of the legs. A CT scan revealed a large appositional thrombus in the distal aorta and a subocclusive thrombus in the left common iliac artery. The rest of the iliac axes were free from thrombus, as were the common and superficial femoral arteries. However, there were bilateral occlusions of the deep femoral arteries as well as from the popliteal arteries downward. Bilateral groin incisions and exposure of the femoral bifurcations were performed. Hypogastric arteries were protected by advancing 24F sheaths into the common iliac arteries. Several thrombectomy maneuvers of the distal aorta and the common iliac arteries were performed using an 8–10F venous Fogarty^®^ catheter. All thrombus material could be retrieved through the DrySeal valves. The control angiogram revealed a dissection at the level of the aortic bifurcation, causing an aorto-caval fistula. This was treated successfully by implanting 8 mm kissing stentgrafts (Gore^®^ Viabahn^®^ VBX balloon expandable stentgrafts (W. L. Gore & Associates, Inc., Flagstaff, AZ, USA)) in both iliac arteries, extending 3 cm into the aorta. Both large sheaths were removed, and the deep femoral, popliteal, tibial, and pedal vessels were thrombectomized surgically. The patient was immediately heparinized therapeutically and subsequently switched to oral anticoagulation. A postoperative CT scan showed patent stentgrafts in the aortic bifurcation and no residual thrombus. Both hypogastric arteries were patent. The patient was discharged home on postoperative day 11 with bilaterally palpable foot pulses.

### 3.4. Patient 4

A 53-year-old morbidly obese female patient was referred for acute ischemia of her left leg. A CT scan demonstrated a subocclusive thrombus of her left common iliac artery as well as an occlusion of the popliteal and all tibial arteries. Iliac thrombectomy was performed using a 5F Fogarty^®^ catheter. Due to a slight downward movement of the sheath during the thrombectomy maneuver, some thrombus occluded the origin of the hypogastric artery. This could also be solved by pulling the sheath back into the external iliac artery, probing the hypogastric artery using a 0.018″ wire, inserting a 3F OTW Fogarty^®^ balloon, and pulling the thrombus back into the sheath. The popliteo-tibial thrombus was removed through conventional below-the-knee access. The leg fully recovered, and the patient was discharged home on postoperative day 6 with palpable foot pulses.

### 3.5. Patient 5

A 53-year-old male patient presented in the emergency room with absolute ischemia of his right leg. A CT scan revealed an occlusion of his right common iliac artery, with the hypogastric, external iliac, and femoral arteries patent but with occlusion of the popliteo-tibial vessels as well. The common iliac artery was thrombectomized with a 5F Fogarty^®^ catheter, protecting the hypogastric artery with a 3F OTW Fogarty^®^ balloon catheter. The popliteo-tibial emboli were removed surgically via a standard below-the-knee approach. A fasciotomy of all four tibial compartments was performed. The patient fully recovered from his limb ischemia. Fasciotomy wounds were closed on postoperative day 10, and the patient was discharged home on postoperative day 17 with palpable foot pulses.

### 3.6. Patient 6

**Figure 3:**
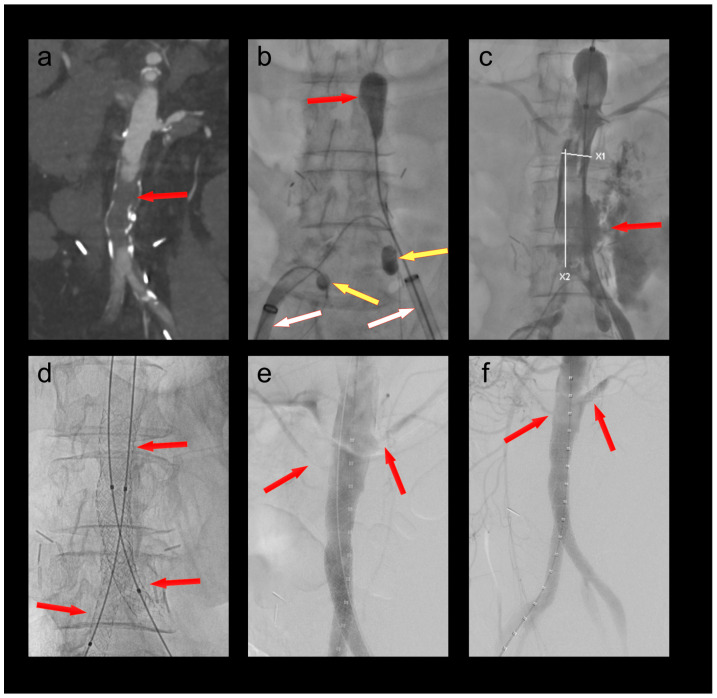
(**a**) Occlusion of the infrarenal aorta (red arrow); (**b**) thrombectomy of the infrarenal aorta (red arrow) under balloon protection of both hypogastric arteries (yellow arrows). The large-bore sheaths are occlusive in both external iliac arteries (white arrows); (**c**) iatrogenic rupture of the infrarenal aorta; (**d**) covered endovascular reconstruction of the aortic bifurcation (red arrows); (**e**) stenoses at the origins of the left renal and a right accessory renal artery (red arrows); (**f**) no residual stenoses after PTA of the right accessory renal and PTA/stenting of the left renal artery (red arrows).

A 68-year-old female patient was admitted to the emergency department with chest pain as well as rest pain in both legs. She was diagnosed with acute ischemia of both legs and an inferior ST-elevation myocardial infarction. CT angiography revealed an occlusion of her distal aorta. After stent PCI of her right coronary artery, she was transferred to the hybrid operating room, where her aorta was thrombectomized with her hypogastric arteries protected by 4F OTW Fogarty^®^ catheters (Figure 3). When passing the thrombectomy balloon through the underlying stenosis in the distal aorta, a rupture of the latter occurred. This was treated by a covered endovascular reconstruction of the aortic bifurcation (CERAB) using Gore^®^ Viabahn^®^ VBX balloon expandable stentgrafts (W. L. Gore & Associates, Inc., Flagstaff, AZ, USA). Furthermore, a near occlusion of her left renal artery was stented, and a near occlusion of her right accessory renal artery was dilated. The patient recovered both from her myocardial infarction and her limb ischemia. She was discharged to cardiac rehabilitation with bilaterally palpable foot pulses.

### 3.7. Patient 7

A 87-year-old female patient was admitted to the emergency department with acute ischemia of her right leg. CT-angiography revealed an occlusion of her common and external iliac arteries. The vessels were patent from the epigastric vessels downward. The etiology of the arterial occlusion was most likely cardio-embolic with newly diagnosed atrial fibrillation. The distal common femoral artery was punctured percutaneously, and an 18F sheath was inserted with its tip remaining distal to the thrombus. Embolectomy was performed over a 5.5F OTW Fogarty^®^ catheter. She had palpable foot pulses at the conclusion of the procedure.

### 3.8. Patient 8

A 65-year-old male patient was admitted with new-onset rest pain and short-distance claudication of his left leg. He had undergone EVAR with bilateral iliac branch devices 6 months prior. A CT scan revealed a complete occlusion of the left EVAR limb and the hypogastric branch of the iliac branch device. The distal part of his external iliac artery was patent. After percutaneous puncture of his left common femoral artery, a 24F sheath was advanced into the left external iliac artery. The EVAR limb was thrombectomized using a 5.5F OTW Fogarty^®^ catheter. Using a steerable sheath, the internal iliac branch was thrombectomized using a 4F OTW Fogarty^®^ catheter. Due to the remaining thrombus, the entire internal iliac bridging Stentgrafts were relined using Gore^®^ Viabahn^®^ VBX balloon expandable stentgrafts. Due to the remaining thrombus in the main EVAR limb, the latter was relined as well using Gore^®^ Viabahn^®^ VBX balloon expandable stentgrafts. Unfortunately, the angiogram revealed an embolus inside the femoral bifurcation as well as contrast dye between the 24F sheath and the walls of the external iliac artery. The sheath had obviously not been entirely occlusive. A cutdown to the femoral bicurcation was performed, the sheath removed, and the puncture site closed. A transverse arteriotomy was performed in the distal common femoral artery, a standard thrombectomy of the femoral bifurcation was performed, and the arteriotomy was closed. Angiography of the leg revealed no peripheral embolization, and the patient had palpable foot pulses.

## 4. Discussion

To our knowledge, the technique that we describe here is novel and has not been published before. It is a technique that combines the advantages of open surgical standard thrombectomy and the minimal invasiveness of endovascular procedures [7], while at the same time avoiding the disadvantages of the respective techniques. In practice, it can be performed purely percutaneously while still providing protection against distal embolization. The technique is extremely flexible, as it can be used with any combination of wires, Fogarty^®^ catheters, and OTW Fogarty^®^ catheters. Therefore, it is suitable for a wide variety of indications, as described in our cases above. Originally, we had the idea of using the technique on a severely ill COVID-19 patient. However, in the meantime, the majority of our patients treated with the technique were not COVID-19 positive and had a variety of indications for their procedures. In practice, while we first applied the technique to patients that had purely thrombotic events in difficult areas of the thoracoabdominal aorta, we have expanded the indications for the technique over time. On the one hand, this includes patients like patient 7, who presented above with an embolic occlusion of her right iliac arteries. This, of course, could have been treated easily by simply performing a cutdown to the groin vessels and performing a simple classic thrombectomy maneuver. In such cases, the advantage of our technique is limited to the absence of a cutdown with potential wound healing complications in the groin. On the other hand, we have also expanded the indication for the technique to patients like patient 6 above, who had an acute occlusion of her distal aorta with an underlying chronic stenosis of the latter. The thrombectomy maneuver resulted in a rupture of the aorta. Of course, it could be argued that she would have been better treated by an aorto-bi-iliac or subclavian-bi-femoral graft. However, the patient had just undergone stent PCI of her right coronary artery and had been loaded with antiplatelet drugs and full anticoagulation, and we wanted to avoid major surgery out of respect for potential bleeding complications. Furthermore, we could not be sure based on the preoperative CT scan whether there was really an underlying chronic stenosis. Last, the treatment of the aortic rupture by covered endovascular reconstruction of the aortic bifurcation highlights the flexibility of our technique even in the presence of complications.

With our technique, there are, in principle, two different mechanisms of protection against embolization during thrombectomy: First, the size of the sheaths used must be chosen based on preoperative imaging in order to deliberately occlude either the common iliac or the external iliac arteries based on the localization of the thrombus/embolus. This is essential to avoid distal embolization of the lower limbs. Second, vessels that cannot be protected by the large-bore sheaths occluding the iliac arteries are instead protected selectively by inserting a wire and inflating an OTW Fogarty^®^ balloon at their origins. This is preferably conducted from the contralateral side of the actual thrombectomy maneuver in order not to pull out the OTW Fogarty^®^ catheters by mistake during thrombectomy. In our experience, we have protected up to four aortic branches in one patient with OTW Fogarty^®^ catheters and did not experience any dislocation of the latter during thrombectomy.

The beauty of this technique, in addition to being minimally invasive while still protecting against embolization, is its almost indefinite flexibility. The large diameters of the sheaths allow for the insertion of several wires and catheters through a single sheath. This in turn allows for the reservation of the second sheath for the thrombectomy itself, which makes this maneuver quite simple. In addition, once the thrombectomy is carried out, additional endovascular procedures can be performed if needed. In our series, additional procedures included the extension of a bridging stentgraft in a renal artery; thrombectomy of a coeliac trunk; kissing stentgrafts of the common iliac arteries; covered endovascular reconstruction of the aortic bifurcation; PTA and stenting of renal arteries; the thrombectomy of a hypogastric artery; and relining of an EVAR limb and an internal iliac branch of an iliac branch device.

One specific complication that we experienced so far, the embolization into a hypogastric artery while trying to have the large-bore sheath occlusive in the distal common iliac artery, could have been prevented by having the sheath in the external iliac artery and securing the ostium of the hypogastric artery with an OTW Fogarty^®^ catheter in the first place, as was conducted in other cases. Therefore, it could be argued that always placing the large-bore sheath in the external iliac artery rather than advancing it into the distal common iliac artery would be sensible. This would also facilitate the choice of sheath sizes because the diameter of the external iliac arteries is usually smaller than that of the common iliac arteries.

While some of the above additional procedures were planned, others became necessary because of complications of the thrombectomy maneuver itself, such as vessel dissections or even ruptures. Therefore, the technique, while minimally invasive, is far from being without complications. While those complications could have occurred during standard classical thrombectomy maneuvers performed through a cutdown in the groins as well, it cannot be ruled out that one subconsciously tends to be more aggressive with balloon inflation knowing that any complication can be visualized and treated without delay compared to the classical thrombectomy maneuver. However, this is speculative. More importantly, if such complications do occur, one is certainly better prepared to cope with them with large sheaths in place compared to a “blind” thrombectomy maneuver through an arteriotomy in the groin.

In one of the nine procedures that we have performed so far, an embolization of the femoral bifurcation occurred during thrombectomy due to a failure of the 24F sheath to entirely occlude the external iliac artery. This was because the diameter of the latter was slightly too big for the biggest available sheath and could (and should) have been prevented by performing a cutdown in the first place and clamping the common femoral artery distal to the sheath. All other complications could be managed endovascularly through the large-bore sheaths, and no conversion to major open surgery was necessary. Even more importantly, all procedures were successful from a clinical standpoint, with all patients having palpable foot pulses at the completion of their procedures. Furthermore, none of our patients required any reinterventions of the target vessels.

Our experience with this novel technique is so far limited to nine procedures. Therefore, it is certainly too early to rule out the possibility of additional complications that have not arisen so far.

Some limitations of our technique merit mentioning: First, the choice of sheaths to be used is limited to Gore^®^ DrySeal because they provide the possibility of actively deflating their valves, which is necessary to remove the thrombus from the sheaths while leaving the sheaths in place. This could in theory be overcome—when using different sheaths—by removing the sheath containing the thrombus, flushing it out on the table, and then reintroducing the sheath. However, this would make the procedure somewhat more complicated. Second, certain endovascular skills are helpful when selectively probing and blocking aorto-iliac branch vessels. In practice, operators experienced with branched and fenestrated EVAR procedures are probably well prepared to perform such procedures. Third, a certain stock of endovascular materials, such as large bore sheaths, different wires, OTW Fogarty^®^ catheters and large thrombectomy catheters, PTA balloons, stents, and stentgrafts, should be available to perform these procedures and cope with complications.

In conclusion, we believe that this novel technique is a very valid addition to the vascular surgeon’s armamentarium when treating aorto-iliac thrombotic events because it is minimally invasive while still protecting against embolization. It also offers the flexibility to perform a wide range of additional endovascular procedures where needed.

## Figures and Tables

**Table 1:** Patient and procedure characteristics.

Patients (*n* = 8)	
Age (median, IQR ^1^)	63 (53; 68.5)
Gender male	4/8 (50%)
COVID-19 positivity	2/8 (25%)
**Procedures (*n* = 9)**	
Size of sheaths ^2^	22F (20F; 24F) (18F; 24F)
Arteries protected by balloons ^2^	0 (1; 2.75) (0; 4)
Complications of the procedures	No: 4/9 (44.4%)
	Yes: 5/9 (55.6%)
	1 dissection of renal artery
	1 dissection of aortic bifurcation
	1 embolization to hypogastric artery
	1 rupture of distal aorta
	1 embolization to femoral bifurcation
Additional endovascular procedures	No: 3/9 (33.3%)
	Yes: 6/9 (66.7%)
	1 relining of RRA bridging stent graft
	1 thrombectomy of coeliac trunk
	1 kissing stentgrafts in aortic bifurcation
	1 thrombectomy of hypogastric artery
	1 CERAB
	1 stenting of renal artery 1 relining of EVAR limb 1 relining of internal iliac branch
Technical success	8/9 (88.9%)
Unplanned cutdown	1/9 (11.1%)
Clinical success	9/9 (100%)

^1^ IQR—interquartile range. ^2^ Due to the low number of procedures, median, IQR, and range are given for these variables.

## Data Availability

Not applicable.
